# Volatile Anästhetika zur präklinischen Analgesie durch Rettungssanitäter – Eine Übersicht

**DOI:** 10.1007/s00101-021-01051-1

**Published:** 2021-10-18

**Authors:** Helmut Trimmel, Alexander Egger, Reinhard Doppler, Christoph Beywinkler, Wolfgang G. Voelckel, Janett Kreutziger

**Affiliations:** 1 Abteilung für Anästhesie, Notfall- und Allgemeine Intensivmedizin, Landesklinikum Wiener Neustadt, Corvinusring 3–5, 2700 Wiener Neustadt, Österreich; 2 Karl Landsteiner Institut für Notfallmedizin, Corvinusring 3–5, 2700 Wiener Neustadt, Österreich; 3 Christophorus Flugrettung, Österreichischer Automobil‑, Motorrad- und Touring Club, ÖAMTC, Baumgasse 129, 1030 Wien, Österreich; 4 Abteilung für Anästhesiologie und Intensivmedizin, Landesklinikum Scheibbs, Eisenwurzenstraße 26, 3270 Scheibbs, Österreich; 5 Abteilung für Innere Medizin, Landeskrankenhaus Rottenmann-Bad Aussee, St. Georgen 2–4, 8786 Rottenmann, Österreich; 6 Abteilung für Anästhesiologie und Intensivmedizin, AUVA Unfallkrankenhaus Salzburg, Dr.-Franz-Rehrl-Platz 5, 5010 Salzburg, Österreich; 7 Paracelsus Medizinische Privatuniversität, Strubergasse 21, 5020 Salzburg, Österreich; 8 Department für Gesundheitsstudien, Universität Stavanger, 4036, 8600 Stavanger, Norwegen; 9 Klinik für Anästhesie und Intensivmedizin, Medizinische Universität Innsbruck, Anichstr. 35, 6020 Innsbruck, Österreich

**Keywords:** Inhalative Analgesie, Rettungsdienst, Methoxyfluran, Prähospitale Schmerztherapie, Rettungsdienstliches Fachpersonal, Inhaled analgesics, Methoxyflurane, Emergency medical service, Prehospital analgesia, Paramedic

## Abstract

Patienten mit Schmerzen können durch den nichtärztlichen Rettungsdienst mitunter nur inadäquat versorgt werden, da aufgrund rechtlicher Einschränkungen die Anwendung stark wirksamer Schmerzmittel (Opioide) bzw. ausbildungsbedingt eine i.v.-Therapie in Deutschland und Österreich oft nicht möglich ist. Häufig müssen Notärzte für schmerzgeplagte Patienten nachgefordert werden, wodurch deren Verfügbarkeit für z. B. vitale Notfälle reduziert sein kann. Inhalativ zu verabreichende Analgetika könnten hierfür eine interessante Alternative darstellen.

Derzeit steht dazu in Deutschland und Österreich Lachgas (N_2_O, als Livopan® im Handel) zur Verfügung, eine Mischung aus jeweils 50 % Lachgas und Sauerstoff. In Österreich ist seit 2018 auch Methoxyfluran (Penthrop®) zur Behandlung mäßiger bis starker Schmerzen nach einem Trauma für die prä- und innerklinische Anwendung bei Erwachsenen zugelassen.

In der Zusammenschau der vorhandenen Literatur, jahrzehntelanger Erfahrung in der Anwendung der Sauerstoff-Lachgas-Mischung im angloamerikanischen Bereich und von inhalativem Methoxyfluran v. a. in Australien sowie aktuellen Studien aus Europa kann gefolgert werden, dass diese bei Einhaltung der Anwendungsvorschriften effektiv, sicher und nebenwirkungsarm sind. Dies bestätigt auch eine eigene Untersuchung zu Methoxyfluran im präklinischen Einsatz. Die Anwendung von Lachgas ist aufgrund des Druckgaszylinders von der Handhabung her etwas aufwendig; Methoxyfluran ist einfacher anzuwenden und bei starken Schmerzen auch wirksamer. Die Zulassung von Methoxyfluran ist jedoch auf Erwachsene beschränkt, wo es mit zunehmendem Alter deutlich besser wirkt. Der Einsatz von Lachgas und insbesondere Methoxyfluran könnte aufgrund der Datenlage wie auch eigener Erfahrungen für rettungsdienstliches Fachpersonal nach entsprechender Einweisung empfohlen werden.

## Abstract (en)

Treatment of acute pain is a central task in emergency medicine. Yet, prehospital pain relief is often insufficient or delayed since the administration of potent intravenous analgesic drugs (such as opioids) is mostly limited to physicians due to legal restrictions or training deficiencies in Germany and Austria. Frequently, prehospitally operating emergency physicians have to be demanded later for anguished patients limiting disposability of physicians for patients who are in a potentially life-threatening condition. Thus, inhaled analgesics could represent an interesting alternative.

A mixture of 50% nitrous oxide and 50% oxygen (N_2_O, Livopan®) has been available in Germany and Austria for several years; however, prehospital use of Livopan has been merely realized and only one trial has been published. In addition, methoxyflurane (Penthrop®), a volatile anesthetic from the group of the dialkyl esters (2-dichloro-1:1-difluoroethyl-methyl-ester) was approved for the treatment of moderate to severe pain following trauma in adults in many European countries in recent years and was brought onto the market in Austria in 2018. Several in-hospital trials demonstrated high effectiveness in this setting.

This article discusses the effects and prehospital areas of application of both substances in the light of the existing literature. We provide a narrative overview of the current study situation and report on a recently performed prehospital application study of methoxyflurane (Penthrop®) from Austria.

The need for pressurized gas cylinders for the use of N_2_O represents a certain limitation in prehospital use. Furthermore, in certain injuries such as of the inner ear or a pneumothorax N_2_O should not be used and the risk of diffusion hypoxemia has to be addressed. Users should be particularly careful and limit the use in alcohol addicts and vegans. The advances of N_2_O are that it is odorless, has a fast onset of action, the usability in patients over 1 month old and has stabilizing effects on the circulation. Plenty of literature regarding prehospital as well as in-hospital use of nitrous oxide in emergency, obstetric and pediatric settings show its effectiveness as a single drug as well as in combination with other analgesics, such as paracetamol or various opioids. Its long tradition in Anglo-American countries is also based on its safety and low rate of side effects.

Methoxyflurane is easier to store and handle and may be slightly more effective in severe pain after trauma; however, its approval is restricted to adults, where it works significantly better with increasing age, based on the declining minimal alveolar concentration (MAC) of all inhaled anesthetics with increasing age. Furthermore, decades of use of inhaled methoxyflurane in Australia have shown the drug is effective, safe and low in side effects and has a broad spectrum of applications. The use of methoxyflurane is limited in patients with severe hepatic or renal insufficiency and the characteristic odor has been described as unpleasant by some patients. In Europe, three large in-hospital trials showed strong pain relief in trauma patients, even comparable to opioids.

Overall, based on the current evidence, the use of nitrous oxide and even more of methoxyflurane may be recommended also for prehospital use by skilled paramedics.

## Hintergrund

Die wirksame Behandlung akuter Schmerzen außerhalb eines Krankenhauses kann für nichtärztliches Rettungsdienstpersonal eine Herausforderung darstellen. Häufig müssen Notärzte für schmerzgeplagte Patienten nachgefordert werden, wodurch deren Verfügbarkeit für z. B. vitale Notfälle limitiert sein kann.

An möglichen Ursachen werden in der Literatur verschiedene Aspekte diskutiert:

- unzureichende Ausbildung bzw. klinische Erfahrung des eingesetzten Personals,
- fehlende Therapiestandards [3, 22],
- Sorge um potenzielle, ggf. nicht-beherrschbare Nebenwirkungen [22, 49],
- gesetzliche Limitationen für das nichtärztliche Rettungsdienstpersonal in Bezug auf die i.v.-Verabreichung von Medikamenten bzw. die Anwendung von Opioiden,
- unzureichende Erfassung (z. B. mittels Numeric Rating Scale [NRS] oder visueller Analogskala [VAS]) und Unterschätzung von Schmerzzuständen im notärztlichen wie auch im nichtärztlichen Bereich [22, 49],
- verzögerter Wirkungseintritt bzw. zu geringe Wirkung von Nicht-Opioidanalgetika bei starken Schmerzen, auch wenn einzelne Untersuchungen gegenteilige Effekte aufzeigen [15, 21, 37].

## Voraussetzungen für nichtärztlich verabreichte Analgesie

Eine adäquate analgetische Versorgung von Notfallpatienten ist prähospital wie innerklinisch ein wichtiges Qualitätskriterium, flächendeckend und ggf. auch arztunabhängig standardisiert sicherzustellen [27]. Dies sollte mit klaren Algorithmen und einfach anzuwendenden, nebenwirkungsarmen Medikamenten auch erreichbar sein. Folgende Voraussetzungen müssen aus Sicht der Autoren dabei erfüllt sein: kein kritischer Einfluss auf Vitalfunktionen (Bewusstsein, Atmung, Kreislauf), milde, einfach beherrschbare Nebenwirkungen ohne Potenzial der Patientengefährdung, einfache Applikation, rascher Wirkungseintritt, gute Steuerbarkeit, einfache Lagerungsfähigkeit (Temperaturstabilität), geringer Dokumentationsaufwand (nicht wie bei Opioiden), geringes Missbrauchspotenzial, akzeptable Kosten. Daneben sind vertretbarer Schulungsaufwand und akzeptable Arbeitsplatzbelastung und schließlich die Freigabe zur Applikation durch Rettungsdienstfachpersonal gemäß nationalen gesetzlichen Festlegungen, wie z. B. dem österreichischen Sanitätergesetz (Bundesgesetzblatt [BGBl.] I Nr. 30/2002) oder dem Notfallsanitätergesetz in Deutschland (BGBl. I S 1384/2014), erforderlich.

## Medikamente zur inhalativen Analgesie

In der Europäischen Union sind aktuell zwei Medikamente, die per inhalationem verabreicht werden und diese Voraussetzungen erfüllen könnten, zugelassen: Lachgas (N_2_O) und Methoxyfluran.

Bei inhalativen Analgetika entfällt naturgemäß die Notwendigkeit eines i.v.-Zugangs für die Schmerzbehandlung, zudem erfolgt die Verabreichung patientengesteuert: Je nach Schmerzniveau kann der Patient die Zufuhr jederzeit unterbrechen oder durch Erhöhung von Atemfrequenz bzw. Atemzugvolumen auch intensivieren. Dies erfordert selbstverständlich ein Minimum an Compliance und Sprachverständnis. Der Wirkungseintritt ist rasch, die Verträglichkeit gut – kritische Nebenwirkungen (anhaltende Bewusstseinstrübung, interventionsbedürftige Atem- oder Kreislaufdepression) sind für beide Substanzen in der prähospitalen Anwendung nicht beschrieben [17, 43, 44].

Die analgetische Potenz von Methoxyfluran scheint im Vergleich zu Opioiden nicht sehr stark: Circa ein Drittel der Potenz von Morphin [40] bzw. 3 ml Methoxyfluran etwa vergleichbar mit ca. 25 µg Fentanyl i.v. [36], wurde in limitierten Settings beschrieben. Trotzdem kann Methoxyfluran in der klinischen Anwendung auch mit Opioiden bestehen [35]. Im angloamerikanischen Raum hat sich die Mischung aus jeweils 50 % Sauerstoff und Lachgas (Entonox®) prähospital wie innerklinisch seit Jahrzehnten bewährt [6, 17] und wird auch in Kombination mit Opioiden angewendet [28, 41, 50]. Die Zulassung in Deutschland und Österreich zur „*Behandlung kurzzeitiger Schmerzzustände von leichter bis mittlerer Intensität*“ gemäß Fachinformation liegt für Patienten jeden Alters > 1 Monat seit Jahren vor. Lachgas ist hier als Livopan® (Fa. Linde Healthcare, 85764 Oberschleißheim bzw. bzw. 1030 Wien, Österreich) im Handel.

Methoxyfluran ist ein volatiles Anästhetikum, welches nur mehr in niedriger, rein analgetischer Konzentration zum Einsatz kommt (in Australien seit 1975, in Neuseeland seit 2002, in einigen Ländern Osteuropas seit 2010) [8, 11, 13, 24]. Die Anwendung von Methoxyfluran ist an mehr als 5 Mio. Patienten erfolgt und an über 200.000 erwachsenen und pädiatrischen Patienten in Registerdaten (Tab. 1) oder prospektiven Studien untersucht [9, 34, 40]. Ernste Nebenwirkungen, insbesondere das Auftreten einer malignen Hyperthermie, wurden bis dato nicht publiziert [32].

**Table 1:** 

Autor	Typ	Setting	Anzahl, Patienten [Methoxyfluran-Medikation]	Alter	Methoxyfluran, Ergebnis
Middleton 2010 [22]	Retrospektiv, Vergleichsstudie	Prähospital, Australien	42.844 [19.235]	16–100 Jahre	3,2 mediane Reduktion in der VNRS-11 (59,1 % erreichten ≥ 30 %ige Reduktion in der Schmerzwahrnehmung)
Johnston 2011 [23]	Retrospektiv, Beobachtungsstudie	Prähospital, Australien	1024 [465]	Keine Einschränkung	VAS-Reduktion: 5 min: 2,0; bei Klinikeinlieferung: 2,5
Oxer 2007 [42]	Retrospektiv, Beobachtungsstudie	Prähospital, Australien	17.334 [10.706]	Keine Einschränkung	> 90 % gute oder mind. teilweise Schmerzerleichterung
Bendall 2011 [18]	Retrospektiv, Vergleichsstudie	Prähospital, Australien	3312 [2093]	5–15 Jahre	Effektive Analgesie bei 78,3 % aller Patienten, (VNRS-11-Reduktion in ≥ 30 %)
Jacobs 2010 [31]	Retrospektiv, Vergleichsstudie	Prähospital, Australien	135.770 [17.629]	1–104 Jahre	Kein Unterschied in kardialen Komplikationen, Nieren- oder Leberfunktionseinschränkungen, Diabetes oder Krebserkrankungen bei Patienten, die Methoxyfluran erhalten haben (Vgl. zu Gesamtkollektiv)

*VNRS* „verbal numeric rating scale“, *VAS* „visual analog scale“

Während sich Lachgas in Mitteleuropa trotz einiger positiver Studien [18, 29] bislang kaum durchgesetzt hat, war Methoxyfluran hier bis vor Kurzem gar nicht zugelassen. Erst 2018 erhielt es die Zulassung durch die European Medicine Agency (EMA); in Österreich wird es unter dem Namen „Penthrop®“ zur Behandlung mittlerer und starker Schmerzen nach Trauma vertrieben. In Deutschland stoppte der Hersteller Mundipharma im Oktober 2019 die Markteinführung von Methoxyfluran („Penthrox®“). Als Gründe wurden gemäß einer Information der Arbeitsgemeinschaft Notärzte in Nordrhein-Westfalen (AGNNW) „*Zulassungs- und Anwendungsbeschränkungen*“ ohne weiterführende Informationen sowie „*Probleme durch den Brexit*“ angegeben. Nach mündlicher Mitteilung seitens der Firma hatte die Entscheidung v. a. Marketinggründe, die ausschließlich Deutschland betrafen. Dabei hatten einige Rettungsdienste bereits die Einführung von Penthrox® geplant [1].

## Literaturüberblick

### Lachgas

#### Pharmakologie

Lachgas wurde erstmals 1772 von Priestley und Leeds synthetisiert, aber erst 1844 als Analgetikum praktisch eingesetzt. In der klinischen Anwendung ist die Farb- und Geruchslosigkeit insbesondere bei Kindern vorteilhaft. Lachgas bewirkt über den N‑Methyl-D-Aspartat(NMDA)-Rezeptor kaum Atemdepressionen, jedoch auch keine suffiziente Anästhesie (minimale alveoläre Konzentration, MAC = 110 %) [33]. Durch die hohe Diffusionskapazität flutet das Gas im Körper extrem schnell an und wieder ab. Bei Anwendung von Konzentrationen >50 % ist die Gabe von 100 %igem Sauerstoff über mindestens 3 min am Ende der Verabreichung notwendig, um eine alveoläre Diffusionshypoxie zu vermeiden. Vorsicht ist weiters bei der notfallmedizinischen Anwendung bei Innenohrverletzungen oder Pneumothorax geboten. Eine leichte Sympathikusstimulation wurde ebenfalls beschrieben, die den gering negativ inotropen Effekt kompensieren kann. Bei kurzfristiger, prähospitaler Anwendung drohen außer Übelkeit, Bewusstseinseinengung und der erwähnten Diffusion in Hohlorgane aufgrund der geringen Metabolisierung (*beachte*: Darmbakterien) keine relevanten Nebenwirkungen. Die Gabe von Antiemetika in niedriger Dosierung, insbesondere 5HT_3_-Antagonisten oder Droperidol (1,25 mg/70 kgKG) kann evtl. erforderlich sein. Hochdosierte Anwendung über einen längeren Zeitraum (> 6 h) kann jedoch zu Störungen im Vitamin‑B_12_-Stoffwechsel führen (Oxidation des Kobaltions im Kobalamin). Vitamin B_12_ ist ein wichtiger Kofaktor der Methioninsynthetase, die wiederum bei der Synthese von DNA, Cholin, Phospholipiden und Myelin mitwirkt. Bei Mangel an Cobalamin drohen daher hämatologische, neurologische und reproduktionstoxische Wirkungen sowie ggf. Störungen der embryonalen Entwicklung. Daher sollte es bei Patienten nach einer Gastrektomie, mit „Blind-loop“-Syndrom, Alkoholismus oder Patienten, die sich streng vegetarisch/vegan ernähren, aufgrund deren eingeschränkter Kobalaminreserven nur kurzfristig angewandt werden.

#### Praxis

Lachgas-Sauerstoff-Mischungen sind seit Jahrzehnten zur Analgesie im Einsatz. Die Literatur ist umfangreich, sowohl für den prä- als auch für den innerklinischen Bereich (Notfallaufnahme, Geburtshilfe, Pädiatrie). Beispielhaft und relativ aktuell sei hier die randomisierte, placebokontrollierte Studie von Ducasse et al. in Toulouse genannt, die die erfolgreiche Anwendung von Lachgas durch Paramedics bei erwachsenen Traumapatienten zeigen konnten. In zwei Drittel der Patienten mit einem initialen Schmerzniveau von 6 auf der NRS wurde eine ausreichende Analgesie (NRS < 3 nach 15 min) erreicht [18]. Auch bei Kindern und Jugendlichen wurde Lachgas, allerdings in Kombination mit intranasalem Fentanyl, für kurzfristige, notfallmedizinische Eingriffe wie Repositionierungen und Lagerungsmanöver erfolgreich eingesetzt [30]. Die Rate an Nebenwirkungen wie tiefere Sedierung oder Übelkeit und Erbrechen war mit < 5 % für ein innerklinisches Setting tolerabel [51]. Auch in Deutschland wurde die Lachgas-Sauerstoff-Mischung bei erwachsenen Traumapatienten (*n* = 35), etwa im Rahmen der LabET-Studie, erfolgreich als Monotherapeutikum eingesetzt und gefolgert, dass dieses Medikament für die Anwendung durch nichtärztliches Rettungsdienstfachpersonal durchaus geeignet wäre [29].

**Figure 1:**
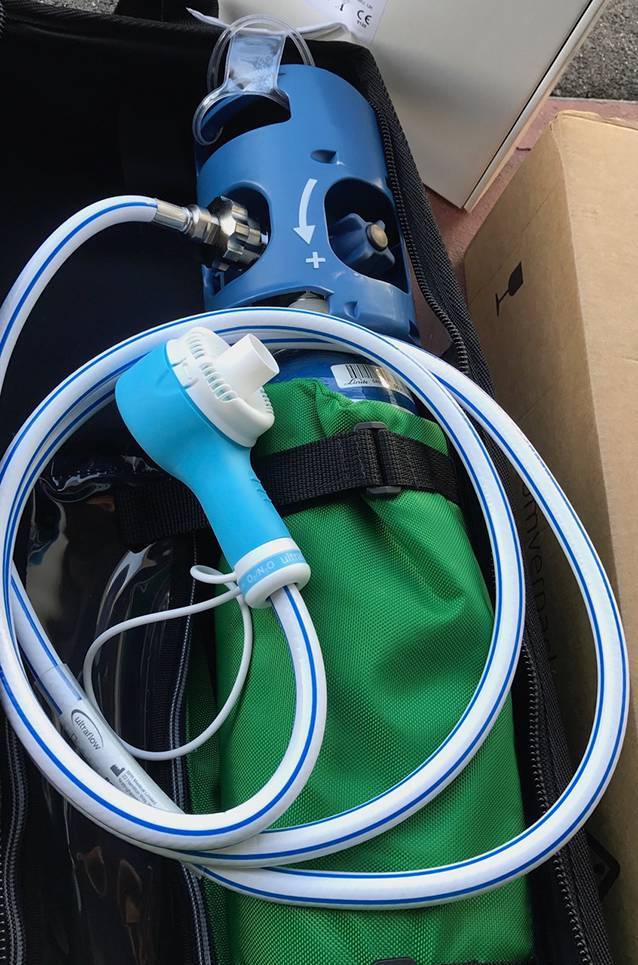

In Österreich wurde Lachgas zur Überbrückung bei stärksten Schmerzen (im Schnitt NRS = 8) 2012 in einem Pilotprojekt des Roten Kreuzes Steiermark durch nichtärztliches Rettungsdienstfachpersonal bis zum Eintreffen des Notarztes in einer kleinen Kohorte von erwachsenen Notfallpatienten (*n* = 65) nach einem Trauma angewandt. Die Schmerzintensität sank binnen 10 min nach Beginn der Applikation von initial 8 Punkten auf der NRS-Skala um 3,4 Punkte. Diese Behandlung wurde von 91 % der Patienten subjektiv als angenehm empfunden (ausreichende Schmerzreduktion), wenngleich ein NRS von 4,6 Punkten nach Therapie aus ärztlicher Sicht nicht zufriedenstellen kann. Ein Nachteil von Lachgas ist jedenfalls das relativ hohe Gewicht der notwendigen Devices – Druckgasflasche, Druckminderer und Demand-Ventil (Abb. 1) beanspruchen relativ viel Platz und lassen N_2_O für manche Bereiche (z. B. alpines Setting) als eher ungeeignet erscheinen.

### Methoxyfluran

#### Pharmakologie

Bereits 1940 wurden fluorierte Kohlenwasserstoffe von Robbins auf ihre anästhesiologische Aktivität hin untersucht [48]. Doch erst 1956 testeten Van Poznak und Artusio 2,2-Dichlor-1:1-difluorethylmethylester (C_3_H_4_Cl_2_F_2_O) aus der Klasse der Dialkylester in Hunden [45]. Am Menschen wurde Methoxyfluran erstmals 1960 untersucht [5, 46]. Die klare, nahezu farblose Flüssigkeit mit charakteristisch fruchtigem Geruch (Fachinformation Penthrop® [26]) hat von allen bisher beschriebenen volatilen Anästhetika die höchste Potenz mit der niedrigsten MAC (0,16 %). In niedriger Dosierung hat es sehr gute analgetische Eigenschaften [52]. Die Metabolisierung findet in der Leber statt, wo es zu Fluorid und Dichloressigsäure demethylisiert (CYP450 2E14) wird, um dann durch Dechlorierung zu Methoxydifluoressigsäure und Oxalsäure abgebaut zu werden. Dabei wird Fluorid freigesetzt, was bei lang dauernder Anwendung in hohen Dosierungen nephrotoxisch wirken kann [16, 38]. Bei kurzer Anwendung im Rahmen von notfallmedizinischen Einsätzen oder Interventionen ist diesbezüglich kein Risiko zu erwarten. Dennoch wird seitens des Herstellers empfohlen, Methoxyfluran bei anamnestischem Hinweis auf vorbestehende Leberschädigung oder klinisch manifester Niereninsuffizienz nicht einzusetzen. Als unerwünschte Wirkungen werden Schwindel, Kopfschmerzen, Schläfrigkeit, Mundtrockenheit und Übelkeit angegeben. Seltener, doch für den Rettungsdienst relevant sind euphorische Stimmungslage, ein Gefühl wie bei Trunkenheit, Dysarthrie, Amnesie, Diplopie, Blutdruckschwankungen ohne Interventionserfordernis (Hypo- und Hypertonie), Hustenreiz und Speichelsekretion. Bei richtiger Anwendung eines Penthrop®-Inhalators mit Aktivkohlekammer zur Absorption des rückgeatmeten Methoxyflurans sollte kaum Methoxyfluran in die Umwelt gelangen. Bei chronischer Exposition, etwa in geschlossenen Räumen, wurde laut Fachinformation ein Anstieg von Leberenzymen, Blutharnstoff-Stickstoff und Serumharnsäure gefunden.

#### Praxis

In *PubMed* finden sich unter den Suchbegriffen „*inhaled methoxyflurane*“ and „*emergency*“ aktuell 55 Studien. Das Medikament ist, wie erwähnt, seit Jahrzehnten im notfallmedizinischen prä- und innerklinischen Einsatz. Millionen von Patienten wurden durch Paramedics prähospital [13, 34, 40] wie auch von Notfallmedizinern innerklinisch erfolgreich und sicher versorgt [14, 25, 43]. Bei über 200.000 Notfallpatienten mit kurzer Anwendung von Methoxyfluran, die in publizierte Studien eingeschlossen wurden, wurde bisher kein einziger Fall von maligner Hyperthermie – um die potenziell gefährlichste Nebenwirkung zu nennen – oder Hinweise auf Nierenschädigung dokumentiert. Auch sonst sind die Nebenwirkungen mild und es ist eigentlich erstaunlich, dass die Anwendung dieser Substanz in Europa bisher nur von begrenztem Interesse war. Mittlerweile liegen für Methoxyfluran neben der Zulassungsstudie („STOP!-Trial“[14]) weitere europäische, prospektive, randomisierte Studien aus Frankreich, Italien und Spanien vor, die in innerklinischen Notfallaufnahmen durchgeführt wurden [12, 39, 47]. Methoxyfluran senkte im Vergleich zu Placebo Schmerzen signifikant – gemessen anhand der VAS (0–100) um durchschnittlich 35 Punkte [14]. In Spanien wurde Methoxyfluran bei posttraumatischen Schmerzen mit der lokal etablierten Standardanalgesie (NSAR bei VAS ≤ 6, darüber ggf. zusätzliche Opioidgaben) multizentrisch verglichen und war dieser nach 20 min deutlich überlegen [12]. Daten aus Notaufnahmestationen in Italien zeigten ebenfalls, dass der analgetische Effekt von Methoxyfluran z. T. hochsignifikant der Standardtherapie überlegen war. Hier wurde Methoxyfluran bei 270 Erwachsenen nach Extremitätentrauma gegen i.v. verabreichtes Paracetamol, Ketoprofen (bei NRS 4–6) und/oder Morphin (bei NRS 7–10) getestet [39]. Auch in Frankreich wurde Methoxyfluran multizentrisch bei Erwachsenen nach Trauma innerklinisch gegen Placebo getestet: Je stärker die Ausgangsschmerzen waren, desto größer war der Effekt von Methoxyfluran. Mit dem inhalativen Analgetikum wurde deutlich weniger Zusatzmedikation in Form von Opioiden benötigt [47].

Nach diesen Erfolg versprechenden innerklinischen Anwendungen stellte sich natürlich die Frage nach der prähospitalen Anwendbarkeit im zentraleuropäischen, notfallmedizinischen Setting. Vor Kurzem wurde in Österreich dazu eine Anwendungsbeobachtung von Methoxyfluran zur prähospitalen Analgesie nach einem Trauma durchgeführt [53]. Methoxyfluran wurde an luft- als auch bodengebundenen Notarztmitteln zur Behandlung von mittleren bis starken Schmerzen bei erwachsenen, bewusstseinsklaren Patienten eingesetzt. Bedingt durch die SARS-CoV-2-Pandemie wurden zwar nur 109 der geplanten 200 Patienten eingeschlossen, dennoch konnten wichtige, z. T. auch signifikante Ergebnisse in Bezug auf Anwenderfreundlichkeit und Effizienz von Methoxyfluran detektiert werden. Die mittels NRS erhobene Schmerzstärke lag bei Eintreffen des Notarztes im Median bei 8 Punkten (7–8), 15 min nach Behandlungsbeginn bei 4 (3–5). Bei knapp zwei Drittel der Patienten wurde keinerlei zusätzliche Analgesie benötigt, wobei sich in der Wirksamkeit eine Altersabhängigkeit zeigte. Je älter die Patienten waren, umso effizienter war Methoxyfluran, d. h., desto seltener wurde eine Zusatzmedikation (bei über 80-Jährigen nur 11 %) benötigt. Dies lässt sich mit der Abhängigkeit des MAC-Werts vom Alter gut erklären und könnte sohin Anlass für Überlegungen zu Dosisvarianzen für verschiedene Altersgruppen geben. Waren Blutdruck und Herzfrequenz der Patienten zu Beginn der Behandlung schmerzbedingt etwas ausgelenkt, normalisierten sich diese unter der Behandlung; Atmung und Bewusstsein wurden nicht beeinträchtigt. Auch die Nebenwirkungen, die in etwa der Hälfte aller behandelten Patienten auftraten, waren in jedem Fall mild. Am häufigsten wurde ein Schwindelgefühl mit 37,8 % angegeben. Mit zunehmendem Alter nahmen die Nebenwirkungen tendenziell in ihrer Häufigkeit, nicht jedoch in der Schwere zu. Unangenehme Geruchs- bzw. Geschmacksempfindungen als Nebenwirkung gaben fünf Patienten an. Ein größeres Problem waren technische Schwierigkeiten: Bei 15 % aller Anwendungen tropfte während der Vorbereitung des Inhalators Flüssigkeit aus dem Mundstück, was eine deutlich verstärkte Geruchsbelastung in der Umgebung zur Folge hatte. In geschlossenen Räumen (Hubschrauberkabine, Rettungswagen) wurde bei jedem fünften Einsatz eine Geruchsbelastung auch ohne das beschriebene Befüllungsproblem rückgemeldet. Dennoch wurde die Anwenderfreundlichkeit in der Summe von den Notärzten als gut bezeichnet und die Zufriedenheit von Patienten und Anwendern war sehr hoch.

## Diskussion

**Figure 2:**
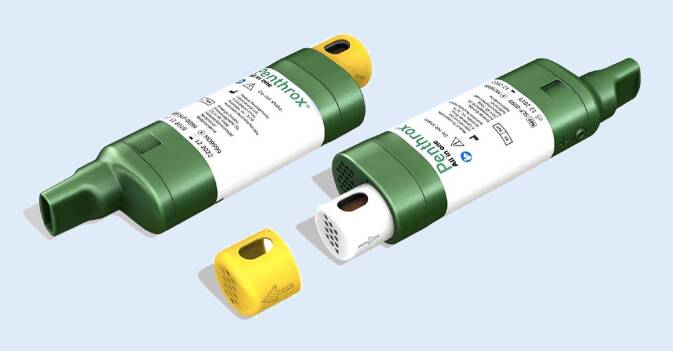

Die Literatur zeigt, dass die inhalative Schmerztherapie nach einem Trauma wie auch bei prozeduralen Schmerzen mit Methoxyfluran ebenso wie mit Lachgas effizient und gut verträglich ist, auch wenn nicht für alle Patienten eine ausreichende Analgesie mit einem inhalativen Analgetikum als Monotherapeutikum erreicht werden kann. Sie zeichnet sich durch einen raschen Beginn der Analgesie aus und wird von den Patienten positiv bewertet [32, 43, 44]. Methoxyfluran ist in der Handhabung deutlich weniger aufwendig als Lachgas [24, 35, 44], wenngleich die Anwendung ebenfalls nicht ganz simpel ist. Der Umgang mit dem Inhalator muss schmerzgeplagten Patienten – insbesondere älteren – einfühlsam erklärt werden: in der Akutsituation ist das nicht immer einfach. Die Wirksamkeit hängt entscheidend davon ab, ob der Patient die Handhabung richtig verstanden hat und durchführt. Die Möglichkeit zur Konzentrationserhöhung durch Verschließen des „*dilutor hole*“ sollte etwa erst nach einer Eingewöhnungsphase angesprochen werden. Patienten können den bei voller Konzentration anfänglich scharfen Geschmack als eher unangenehm empfinden und dann eine weitere Anwendung möglicherweise ablehnen. Nach fünf bis sechs tiefen Atemzügen tritt dieses Phänomen kaum mehr auf, die Analgesie hingegen setzt bereits ein. Auch das Vorbereiten des Inhalators ist nicht ganz einfach: die Flüssigkeit kann beim Befüllungsvorgang aus dem Mundstück auf den Boden tropfen. Dies kann die Wirkung abschwächen bzw. die Nutzungsdauer verkürzen. Hier soll eine Neukonfektion des Devices Verbesserung bringen (Abb. 2: „Penthrox® Inhaler Selfie“, Medical Developments International Limited [MDI], Scoresby, Victoria, Australia). Der neue Inhalator wird die Wirkstoffphiole bereits integriert haben, sodass ein manuelles Befüllen nicht mehr erforderlich ist.

Negative Auswirkungen für das Personal sind im prähospitalen Einsatz weder für Lachgas noch für Methoxyfluran zu erwarten. Die gesetzlichen Grenzwerte für Lachgas sind in den Technischen Regeln für Gefahrstoffe (TRGS) in Deutschland durch die Bundesanstalt für Arbeitsschutz und Arbeitsmedizin [7] bzw. das Bundesministerium für Arbeit in Österreich [10] mit 100 ppm als Tagesmittelwert festgelegt. Kurzfristige höhere Konzentrationen bis 400 ppm werden akzeptiert. Für Methoxyfluran werden in Deutschland und Österreich 2 ppm als Tagesmittelwert (bzw. 14 mg/m^3^) und 4 ppm als kurzfristige Belastung in der Umgebungsluft als Grenzwert festgelegt [2, 10]. Eine aktuelle Studie aus Frankreich, die körpernahe Konzentrationen von Methoxyfluran bei Pflegepersonal einer innerklinischen Notfallstation mit regelmäßiger Anwendung während der 8 h-Schichten analysierte, fand eine mit 0,017 ppm sehr niedrige Durchschnittsbelastung [19]. Auch Messungen der Konzentration von Methoxyfluran in mobilen Ambulanzen konnte keine bedenklichen Konzentrationen beim behandelnden, nichtärztlichen Personal nachweisen (< 0,2 ppm). Die Schwelle für eine Geruchswahrnehmung von Methoxyfluran liegt weit unter den gesetzlichen Grenzwerten, aber über der durchschnittlichen Konzentration in Rettungsfahrzeugen und Ambulanzen (0,13–0,19 ppm) [4, 20]. In der Summe lässt sich trotz des wahrnehmbaren Geruchs von Methoxyfluran bzw. der nichtwahrnehmbaren Luftkonzentration von Lachgas selbst bei regelmäßigem Gebrauch als Notfallanalgetikum keine gesundheitliche Gefährdung der Behandler ableiten.

**Figure 3:**
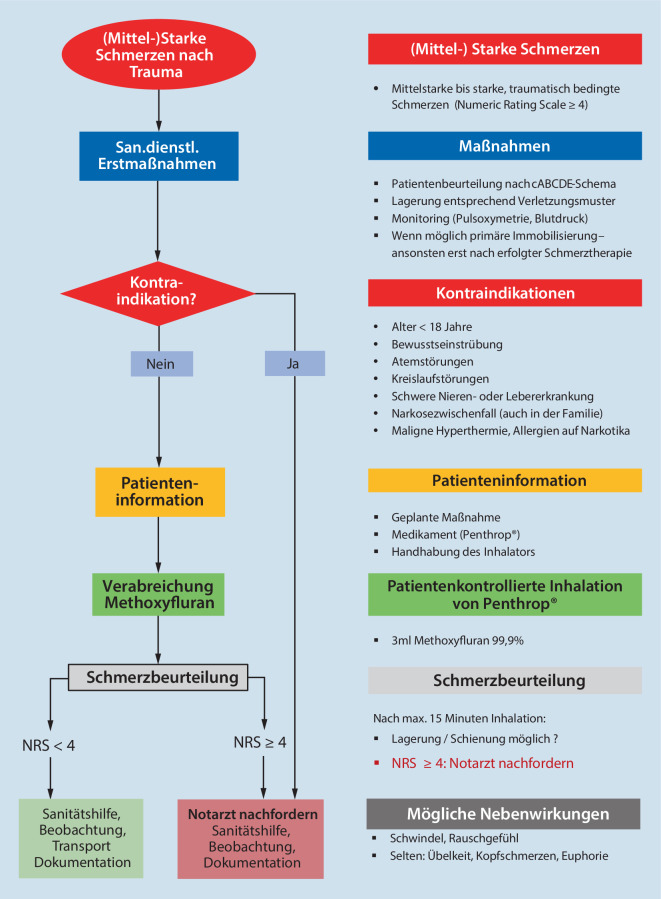

Die Bereitschaft von Notärzten, ein inhalatives Analgetikum einzusetzen, ist angesichts moderner, intranasal oder bukkal appliziert rasch wirksamer Opioide wohl limitiert. Natürlich ist Methoxyfluran weniger wirksam als diese [40]; dieser Umstand muss jedoch gegen die aufwendigere Logistik, das Erfordernis einer exakten ärztlichen (!) Verabreichungsdokumentation, die strenge Überwachung der Lagerhaltung sowie v. a. gesetzliche Limitierungen der Anwendung von Opioiden durch nichtärztliche Berufsgruppen abgewogen werden. Notärzten stehen potente(re) Analgetika zur Verfügung: Unsere Ergebnisse wie auch die vorhandene Literatur bestätigen jedoch, dass Methoxyfluran auch rettungsdienstlichem Fachpersonal in Europa eine suffiziente und v. a. sichere Behandlung von Patienten mit mittleren bis starken Schmerzen ermöglichen kann; ggf. auch in Kombination mit i.v. zu verabreichenden Nichtopioidanalgetika. Unter Anwendung eines definierten Behandlungsalgorithmus (Abb. 3) ließe sich die Zahl notärztlicher Einsätze ausschließlich zur Analgesie verringern und deren Verfügbarkeit für andere, vitalgefährdete Patienten erhöhen [23].

## Fazit für die Praxis

- Lachgas und Methoxyfluran sind sicher anzuwendende, gut wirksame Inhalationsanalgetika. Lachgas ist für alle Altersgruppen, Methoxyfluran derzeit nur für erwachsene Patienten zugelassen, wobei ältere Patienten in der aktuellen Einheitsdosierung deutlich stärker von seiner analgetischen Wirkung profitieren.
- Die Applikation beider Medikamente erfolgt patientengesteuert; die unerwünschten Arzneimittelwirkungen bei kurzer Anwendung sind mild. Besondere Vorteile von Methoxyfluran liegen in der einfachen Logistik und stärkeren Analgesie; Limitationen in der Anwendung können aufgrund unzureichender Compliance auftreten. Ein weiterer Nachteil ist die Geruchsbelastung für das Rettungsdienstpersonal in geschlossenen Räumen, auch wenn dies nie die gesetzlich zulässigen Arbeitsplatzkonzentrationen erreicht. Hier wird herstellerseitig Abhilfe durch einen neuen Inhalator versprochen.
- Die Anwendung von Methoxyfluran anhand eines definierten Algorithmus kann aus unserer Sicht auch für nichtärztliches Personal empfohlen werden.

## References

[1] AGNNW (2019) Penthrox: Einführung auf den deutschen Markt durch Hersteller gestoppt. https://www.agnnw.de/?p=4443. Zugegriffen: 16. März 2021

[2] Ausschuß für Gefahrstoffe (AGS) (2004) Grenzwerte in der Luft am Arbeitsplatz. https://www.umwelt-online.de/recht/t_regeln/trgs/trgs900/900y2000.htm. Zugegriffen: 16. März 2021

[3] Albrecht E, Taffe P, Yersin B (2013). Undertreatment of acute pain (oligoanalgesia) and medical practice variation in prehospital analgesia of adult trauma patients: a 10 yr retrospective study. Br J Anaesth.

[4] Allison SJ, Docherty PD, Pons D, Chase JG (2011) Exposure to methoxyflurane: low-dose analgesia and occupational exposure. https://ajp.paramedics.org/index.php/ajp/article/view/712. Zugegriffen: 16. März 2021

[5] Artusio JF, Poznak AV, Hunt RE (1960). A clinical evaluation of methoxyflurane in man. Anesthesiology.

[6] Baskett PJ (1970). Use of entonox in the ambulance service. BMJ.

[7] Ausschuss für Gefahrstoffe (AGS) (2027) Arbeitsplatzgrenzwerte. https://www.baua.de/DE/Angebote/Rechtstexte-und-Technische-Regeln/Regelwerk/TRGS/pdf/TRGS-900.pdf?__blob=publicationFile&v=18. Zugegriffen: 16. März 2021

[8] Bendall JC, Simpson PM, Middleton PM (2011). Prehospital analgesia in New South Wales, Australia. Prehosp Disaster med.

[9] Bendall JC, Simpson PM, Middleton PM (2011). Effectiveness of prehospital morphine, fentanyl, and methoxyflurane in pediatric patients. Prehosp Emerg Care.

[10] BGBl (2011) MAK-Werte und TRK-Werte. https://www.ris.bka.gv.at/Dokumente/BgblAuth/BGBLA_2007_II_243/COO_2026_100_2_366060.pdfsig. Zugegriffen: 16. März 2021

[11] Blair HA, Frampton JE (2016). Methoxyflurane: a review in trauma pain. Clin Drug Investig.

[12] Borobia AM, Collado SG, Cardona CC (2020). Inhaled methoxyflurane provides greater analgesia and faster onset of action versus standard analgesia in patients with trauma pain: inMEDIATE: a randomized controlled trial in emergency departments. Ann Emerg Med.

[13] Buntine P, Thom O, Babl F (2007). Prehospital analgesia in adults using inhaled methoxyflurane. Emerg Med Australas.

[14] Coffey F, Wright J, Hartshorn S (2014). STOP!: a randomised, double-blind, placebo-controlled study of the efficacy and safety of methoxyflurane for the treatment of acute pain. Emerg Med J.

[15] Craig M, Jeavons R, Probert J, Benger J (2012). Randomised comparison of intravenous paracetamol and intravenous morphine for acute traumatic limb pain in the emergency department. Emerg Med J.

[16] Dayan A (2016). Analgesic use of inhaled methoxyflurane. Hum Exp Toxicol.

[17] Donen N, Tweed WA, White D (1982). Pre-hospital analgesia with entonox. Can Anaesth Soc J.

[18] Ducassé J-L, Siksik G, Durand-Béchu M (2013). Nitrous oxide for early analgesia in the emergency setting: a randomized, double-blind multicenter prehospital trial. Acad Emerg Med.

[19] Frangos J, Belbachir A, Dautheville S (2020). Non-interventional study evaluating exposure to inhaled, low-dose methoxyflurane experienced by hospital emergency department personnel in France. BMJ Open.

[20] Frangos J, Mikkonen A, Down C (2016). Derivation of an occupational exposure limit for an inhalation analgesic methoxyflurane (Penthrox®). Regul. Toxicol. Pharmacol..

[21] Friday JH, Kanegaye JT, McCaslin I (2009). Ibuprofen provides analgesia equivalent to acetaminophen-codeine in the treatment of acute pain in children with extremity injuries: a randomized clinical trial. Acad Emerg Med.

[22] Galinski M, Hoffman L, Bregeaud D (2018). Procedural sedation and analgesia in trauma patients in an out-of-hospital emergency setting: a prospective multicenter observational study. Prehosp Emerg Care.

[23] Galinski M, Ruscev M, Gonzalez G (2010). Prevalence and management of acute pain in prehospital emergency medicine. Prehosp Emerg Care.

[24] Gaskell AL, Jephcott CG, Smithells JR, Sleigh JW (2016). Self-administered methoxyflurane for procedural analgesia: experience in a tertiary Australasian centre. Anaesthesia.

[25] Gillis M, Keirens A, Steinkamm C (2008). The use of methoxyflurane (penthrox) in the emergency department: 411. Reg Anesth Pain Med.

[26] Grindlay J, Babl FE (2009). Review article: efficacy and safety of methoxyflurane analgesia in the emergency department and prehospital setting. Emerg Med Australas.

[27] Häske D, Schempf B, Gaier G, Niederberger C (2014). Prehospital analgesia performed by paramedics: quality in processes and effects under medical supervision. Anaesthesist.

[28] Heinrich M, Menzel C, Hoffmann F (2015). Self-administered procedural analgesia using nitrous oxide/oxygen (50:50) in the pediatric surgery emergency room: effectiveness and limitations. Eur J Pediatr Surg.

[29] Hengefeld N, Lukas RP, Klaus S, Van Aken H, Boh A (2014). Lachgas-Sauerstoff-Gemisch (Livopan®) bei Extremitätentrauma – die LAbET-Studie. Anasth Intensivmed.

[30] Hoeffe J, Trottier ED, Bailey B (2017). Intranasal fentanyl and inhaled nitrous oxide for fracture reduction: the FAN observational study. Am J Emerg Med.

[31] Jacobs IG (2010). Health Effects of Patients Given Methoxyflurane in the Pre-Hospital Setting: A Data Linkage Study. Open Emerg Med J.

[32] Jephcott C, Grummet J, Nguyen N, Spruyt O (2018). A review of the safety and efficacy of inhaled methoxyflurane as an analgesic for outpatient procedures. Br J Anaesth.

[33] Jevtović-Todorović V, Todorovć SM, Mennerick S (1998). Nitrous oxide (laughing gas) is an NMDA antagonist, neuroprotectant and neurotoxin. Nat Med.

[34] Johnston S, Wilkes GJ, Thompson JA (2011). Inhaled methoxyflurane and intranasal fentanyl for prehospital management of visceral pain in an Australian ambulance service. Emerg Med J.

[35] Kinsella J, Glavin R, Reid WH (1988). Patient-controlled analgesia for burn patients: a preliminary report. Burns Incl Therm Inj.

[36] Lenz H, Høiseth LØ, Comelon M (2021). Determination of equi-analgesic doses of inhaled methoxyflurane versus intravenous fentanyl using the cold pressor test in volunteers: a randomised, double-blinded, placebo-controlled crossover study. Br J Anaesth.

[37] Luiz T, Scherer G, Wickenkamp A (2015). Prehospital analgesia by paramedics in Rhineland-Palatinate : feasability, analgesic effectiveness and safety of intravenous paracetamol. Anaesthesist.

[38] Mazze RI, Raja SN (2006). Methoxyflurane revisited. Anesthesiology.

[39] Mercadante S, Voza A, Serra S (2019). Analgesic efficacy, practicality and safety of inhaled methoxyflurane versus standard analgesic treatment for acute trauma pain in the emergency setting: a randomised, open-label, active-controlled, multicentre trial in Italy (MEDITA). Adv Ther.

[40] Middleton PM, Simpson PM, Sinclair G (2010). Effectiveness of morphine, fentanyl, and methoxyflurane in the prehospital setting. Prehosp Emerg Care.

[41] Míguez MC, Ferrero C, Rivas A (2019). Retrospective comparison of intranasal fentanyl and inhaled nitrous oxide to intravenous ketamine and midazolam for painful orthopedic procedures in a pediatric emergency department. Pediatr Emer Care.

[42] Oxer HF, Wilkes G (2007). Methoxyflurane is a safe, easy, effective analgesic for prehospital pain relief. Prehosp Disast Med.

[43] Porter KM, Dayan AD, Dickerson S, Middleton PM (2018). The role of inhaled methoxyflurane in acute pain management. Open Access Emerg Med.

[44] Porter KM, Siddiqui MK, Sharma I (2018). Management of trauma pain in the emergency setting: low-dose methoxyflurane or nitrous oxide? A systematic review and indirect treatment comparison. J Pain Res.

[45] Poznak AV, Artusio JF (1960). Anesthetic properties of a series of fluorinated compounds I. Fluorinated hydrocarbons. Toxicol. Appl. Pharmacol..

[46] Poznak AV, Ray BS, Artusio JF (1960). Methoxyflurane as an anesthetic for neurological surgery. J Neurosurg.

[47] Ricard-Hibon A, Lecoules N, Savary D (2020). Inhaled methoxyflurane for the management of trauma related pain in patients admitted to hospital emergency departments: a randomised, double-blind placebo-controlled trial (PenASAP study). Eur J Emerg Med.

[48] Robbins BH (1940). Preliminary studies of anesthetic activity of luorinated hydrocarbons. J Pharmacol Exp Ther.

[49] Scholten AC, Berben SAA, Westmaas AH (2015). Pain management in trauma patients in (pre)hospital based emergency care: current practice versus new guideline. Injury.

[50] Seiler M, Landolt MA, Staubli G (2019). Nitrous oxide 70 % for procedural analgosedation in a pediatric emergency department-with or without intranasal fentanyl?. Pediatr Emer Care.

[51] Seith RW, Theophilos T, Babl FE (2012). Intranasal fentanyl and high-concentration inhaled nitrous oxide for procedural sedation: a prospective observational pilot study of adverse events and depth of sedation. Acad Emerg Med.

[52] Tomi K, Mashimo T, Tashiro C (1993). Alterations in pain threshold and psychomotor response associated with subanaesthetic concentrations of inhalation anaesthetics in humans. Br J Anaesth.

[53] Trimmel H, Egger A, Doppler R et al Usability and efficacy of inhaled methoxyflurane for pre-hospital analgesia—a prospective, observational study. BMC Emerg Med 10.1186/s12873-021-00565-6 PMC8760876 35033003

